# Video-Assisted Transcervical-Transtracheal Repair for Post-Intubation Tracheal Laceration

**DOI:** 10.1093/icvts/ivag191

**Published:** 2026-07-02

**Authors:** Chun-Han Shih, Chien-Wei Huang, Ting-Shou Chang

**Affiliations:** Department of Otolaryngology-Head & Neck Surgery, Kaohsiung Veterans General Hospital, Kaohsiung 813414, Taiwan; Department of Otolaryngology-Head & Neck Surgery, Kaohsiung Veterans General Hospital, Kaohsiung 813414, Taiwan; Department of Otolaryngology-Head & Neck Surgery, Kaohsiung Veterans General Hospital, Kaohsiung 813414, Taiwan; School of Medicine, National Defense Medical University, Taipei 114201, Taiwan; Department of Early Childhood Care and Education, Cheng Shiu University, Kaohsiung 833301, Taiwan

**Keywords:** post-intubation tracheal laceration, tracheal injury, subcutaneous emphysema, iatrogenic airway injury, tracheal repair, transtracheal approach, video-assisted surgery

## Abstract

**Background:**

Post-intubation tracheal laceration (PITL) is a rare but life-threatening complication. Early recognition and appropriate management are critical to prevent severe morbidity.

**Case Presentation:**

A 30-year-old female with class III obesity (BMI: 41.8 kg/m²) presented with progressive subcutaneous emphysema and pneumomediastinum from a 3 cm full-thickness posterior tracheal laceration (Cardillo level IIIa) after orthopaedic surgery under general anaesthesia. On postoperative day 3, she underwent video-assisted transcervical-transtracheal repair via a temporary cervical tracheostomy. Utilizing a rigid endoscope and a protocol-driven, SpO_2_-guided intermittent apnoeic technique, the laceration was successfully repaired with a 3-0 Monocryl running suture without complications.

**Conclusions:**

The video-assisted transcervical-transtracheal approach offers a minimally invasive and effective alternative to conventional surgeries for PITL.

## INTRODUCTION

Post-intubation tracheal laceration (PITL) is rare but can be life-threatening. Patients may present with subcutaneous emphysema, dyspnoea, or may sometimes be asymptomatic.^1^^,^^2^ Early detection and management are essential to reduce complications and mortality rates. In this report, we present a case of significant subcutaneous emphysema after elective intubation, highlighting the importance of early recognition and treatment options in such cases. This study was approved by the Institutional Review Board (IRB No. KSVGH25-CT9-11).

## CASE PRESENTATION

A 30-year-old female with class III obesity (BMI: 41.8; 168 cm, 118 kg) underwent orthopaedic surgery under general anaesthesia with endotracheal intubation. Postoperatively, she complained of persistent chest tightness. On postoperative day 1, physical examination revealed significant swelling and crepitus of the face, neck, and anterior chest wall. Computed tomography (CT) demonstrated extensive air from the trachea to the lower neck and mediastinum, along with marked subcutaneous emphysema in the anterior chest wall (**Figure 1**). Endoscopic evaluation revealed a 3 cm laceration on the posterior tracheal wall, approximately 8 cm below the vocal cords, causing significant air leakage (**Figure 2**).

**Figure 1.**
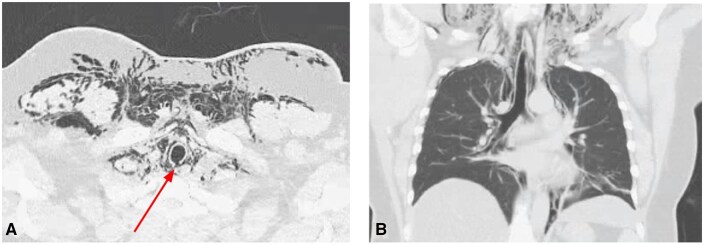
(A, Axial; B, Coronal) CT Scan Showing a Posterior Tracheal Laceration With Subcutaneous Emphysema and Air Tracking to the Lower Neck and Mediastinum, Without Mediastinitis.

**Figure 2.**
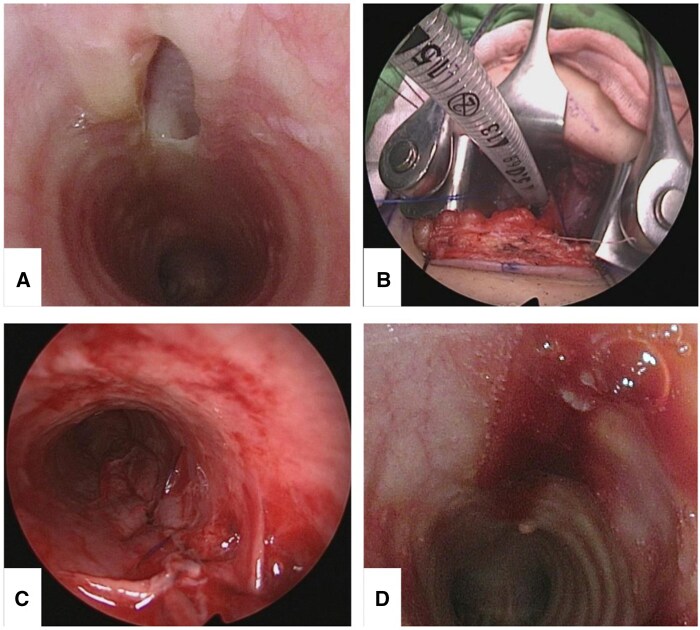
(A) A 3 cm Full-Thickness Laceration Located 8 cm Below the Vocal Cords. (B) Cervical tracheostomy. (C) The laceration was sutured. (D) Healed wound on follow-up.

Because chest pain and subcutaneous emphysema worsened, raising concerns for airway compromise, surgical intervention was arranged on postoperative day 3. First, a cervical tracheostomy was performed. A 6.0-mm armoured endotracheal tube was then inserted through the tracheostomy site, with the cuff placed distal to the tracheal laceration to maintain adequate ventilation. Second, we started the video-assisted procedure. To achieve optimal visualization, 5 mm rigid endoscopes (0° and 30°) were introduced through the same tracheostomy site, which revealed the posterior wall laceration extending between the fifth and seventh tracheal rings. The endotracheal tube had to be intermittently removed, placing the patient in a temporarily apnoeic state for evaluation and suturing. Our protocol was SpO2-guided: when oxygen saturation dropped below 85%, the tube was reinserted for ventilation; once it recovered to above 97%, the tube was removed to resume the procedure.

Under this intermittent apnoeic technique, the laceration was subsequently repaired using a Wandler laryngeal needle holder, and the wound was closed with a 3-0 Monocryl running suture. After the laceration was repaired, a tracheostomy tube was placed. We utilized a flexible fibrescope to confirm that the balloon bypassed the repaired lesion. Postoperatively, due to the patient’s severe obesity and the need for continued mechanical ventilation, a tracheostomy tube was left in place to secure the airway. Follow-up showed resolution of the subcutaneous emphysema, so we removed the tracheostomy tube on postoperative day 16. After 1 month, during outpatient endoscopic evaluations, we noticed good healing of the posterior tracheal wound.

## DISCUSSION

Post-intubation tracheal laceration is very rare and is estimated to be around 0.005%, with approximately 15% in emergency intubations.^1–3^ The most common injury site is the pars membranacea of the cervicothoracic trachea at the midline.^1^^,^^2^

Some risk factors have been reported, including multiple intubation attempts, clinician inexperience, and protrusion of endotracheal tube introducers. Patient-related risk factors include congenital tracheal abnormalities, advanced age, female gender, and bronchial inflammation.^4^ Clinical manifestations may be subtle. Common symptoms include subcutaneous emphysema, dyspnoea of varying severity, and possible pneumothorax or pneumomediastinum.^2^

Diagnosis is based on scope and image. Bronchoscopy should be performed to carefully assess the length and depth of the lesion. Chest X-ray is useful for ruling out pneumothorax and massive pneumomediastinum. Contrast-enhanced CT provides more detailed information, such as small subcutaneous emphysema and tracheal lacerations with adjacent tissue injuries.

The key factor in treatment selection is the depth of the tracheal injury rather than its length.^1^ In our case, a full-thickness tracheal laceration was noted, accompanied by subcutaneous and mediastinal emphysema and mild oesophageal herniation, but without evidence of mediastinitis. According to Cardillo’s revised morphologic classification, this corresponded to a level IIIa tracheal injury.^1^ Conservative treatment or surgery may be considered for this type of patient.^1^^,^^2^

In our case, surgical intervention became necessary because of clinical deterioration. Surgical choices include thoracotomy for injuries in the lower one-third of the trachea, cervicotomy for the upper two-thirds, or video-assisted techniques.^1^^,^^2^ We opted for the video-assisted transcervical-transtracheal approach to repair the posterior tracheal wall laceration.^2^ Compared with the transoral route, this approach shortened the distance to the lower tracheal lesion, significantly improving instrument manoeuvrability for suturing. Furthermore, utilizing the tracheostomy for intermittent intubation avoids the risks of vocal cord injury and prolonged apnoea associated with repeated transoral reintubation. This is a relatively minimally invasive surgical approach, and our subsequent follow-up showed that her conditions recovered very well. The disadvantage of this method is that it is more time-consuming, primarily due to drops in the patient’s blood oxygen levels during the procedure, which necessitate pauses until stabilization. Other potential complications of this technique include intermittent hypoxaemia during the apnoeic periods, bleeding, and the risk of recurrent subcutaneous emphysema if the seal is incomplete. However, carefully coordinating with the anaesthesiologist and utilizing the tracheostomy for direct access and postoperative airway protection can effectively lower these risks.

## CONCLUSION

This case highlights the importance of early recognition and intervention for PITL. Video-assisted repair offers a minimally invasive yet effective surgical option for posterior tracheal wall lacerations, especially in cases with exacerbated subcutaneous and mediastinal emphysema. While the technique may be time-consuming due to intermittent hypoxaemia, its advantages in preserving tracheal integrity and promoting rapid recovery make it a valuable approach.

## Data Availability

The data underlying this article are available in the article and in its online supplementary material.
